# Clinicopathological Features, Treatment Strategy, and Prognosis of Primary Non-Hodgkin's Lymphoma of the Duodenum: A SEER Database Analysis

**DOI:** 10.1155/2020/9327868

**Published:** 2020-01-13

**Authors:** Guoliang Zheng, Yue Wang, Yan Zhao, Zhichao Zheng

**Affiliations:** Department of Gastric Surgery, Cancer Hospital of China Medical University (Liaoning Cancer Hospital and Institute), No. 44 Xiaoheyan Road, Dadong District, Shenyang, Liaoning 110042, China

## Abstract

**Objective:**

Primary duodenum lymphoma (PDL) is extremely rare with limited data available in the literature. In this study, we sought to describe clinical features and identify factors affecting survival in patients with PDL using a large population cohort.

**Methods:**

The Surveillance, Epidemiology, and End Results (SEER) database was queried from 1998 to 2015.

**Results:**

A total of 1060 cases of PDLs were identified. Clinicopathological features as well as survival data of PDLs were analyzed and compared with 10573 primary gastric lymphomas (PGLs) and 3239 primary small intestinal lymphomas (PSILs) from the SEER database. PDL patients were younger in age (60.96 ± 15.205), and the proportion of stage I (53.21%) was higher in Ann Arbor staging. The proportion of PDLs treated by surgery (8.68%) is the lowest among PDLs, PGLs, and PSILs. The DSS of PDLs were significantly better than those of PGLs and PSILs, respectively (10-year survival rate: 21.24% vs. 20.40%, *P*=0.027; 10-year survival rate: 21.24% vs. 16.79%, *P*=0.001). Age, gender, Ann Arbor staging, and histological type were regarded as independent prognostic factor for the DSS by multivariate analysis (all *P* < 0.05). Patients with <65 years, female, stage I, and FL were found to be significantly associated with good DSS. The treatment modality (surgery vs. conservative treatment) was not statistically related to DSS. The proportion of PDL patients who received surgical treatment gradually decreased from 15.60% in period 2 to 5.26% in period 4.

**Conclusions:**

The clinicopathologic features of duodenal lymphoma were significantly different from those of gastric lymphoma and small intestinal lymphoma. The prognosis of PDLs was significantly better than those of the other two groups, and there was no statistical survival benefit from surgery in PDLs.

## 1. Introduction

The most predominant extranodal site in non-Hodgkin's lymphoma (NHL) is the gastrointestinal (GI) tract [1], accounting for 5% to 20% of all NHL cases and 30% to 45% of all extranodal cases [2]. The lesion can occur in any part of the digestive tract from the mouth to the anus, of which stomach is the most common pathogenic sites (60%–75%) [3].

As primary duodenum lymphomas (PDLs) are exceedingly rare, the current researches about PDLs are based on anecdotal reports [4–23]. In the present study, we retrospectively reviewed the clinical and pathological manifestations of lymphomas of duodenum lymphoma for cases based on the largest sample size so far to identify prognostic factors and to clarify the value of treatment modalities in the management of these malignancies.

## 2. Materials and Methods

### 2.1. Data Source and Patient Selection

We queried the SEER database (SEER, 18 November 2017) with SEER Stat version 8.3.5 software to identify 14872 patients who were diagnosed with lymphoma from 1998 to 2015, including 1060 PDLs, 10573 primary gastric lymphomas (PGLs), and 3239 primary small intestinal lymphomas (PSILs). The codes used for lymphoma in the coding system of the International Classification of Diseases for Oncology (ICD-O)-3 were 9590–9729. The search was limited to adult patients (≥18 years old) with the type of follow-up equal to “active follow-up.” The exclusion criteria were as follows: (i) patients without definitely histological confirmation; (ii) patients with only autopsy or death certificate records; (iii) patients with incomplete survival data and follow-up information; (iv) patients without Ann Arbor stage record; and (v) patients without the information of surgery. After screening, we got a total of 10321 patients.

Clinical and pathological variables (e.g., age, gender, race, sex, age at diagnosis, marital status, year of diagnosis, histological type, Ann Arbor stage, whole body symptom of lymphoma based on the AJCC (6th edition) staging system, treatment modalities employed and information of “cause of death and follow-up,” and “multiple primary field”) were extracted from the SEER.

Since the SEER cause-specific death classification variable is defined by taking into account cause of death in conjunction with sequence of tumor occurrence (ie, only one tumor or the first of multiple tumors) and comorbidities (e.g., AIDS and/or site-related diseases), we excluded the patients except that lymphoma was the only one primary cancer or the 1st cancer of 2 or more primaries to avoid the ambiguity of the lymphoma-specific survival [24].

The survival data were available in the measurement unit of months, without precise days. Considering the preconditions that no precise survival days were available and that patients with only autopsy or death certificate records were excluded, a survival time of 0 months was recorded as 0.5 months to include patients who died within 1 month of diagnosis but who did not reach the 1-month threshold [25, 26].

Since this study only involves analysis of the publically available database (SEER) and does not contain any identifying patient information, the ethical approval of this study by the institutional review board (IRB) is not required.

### 2.2. Statistical Analyses

Statistical analyses were performed using the statistical software SPSS 22.0 for Apple (SPSS Inc., Chicago, IL). Numerical variables were expressed as mean ± SD and were analyzed by the *t*-test. Discrete variables were analyzed using the chi-square test or Fisher's exact test. Risk factors for survival were identified by univariate analysis, and COX regression was employed for multivariate analysis. Disease-specific survival (DSS) was analyzed by the Kaplan–Meier method and differences between the curves were compared using the log-rank test. All *P* values were two-sided, and *P* values < 0.05 were considered statistically significant.

## 3. Results

### 3.1. Baseline Demographic Characteristics

Clinical and pathological features of primary duodenum lymphoma (PDL) are summarized in Table 1. In total, 1060 eligible PDL patients were recognized during the 18-year study period (between 1998 and 2015). There was no obvious sex trend: 604 were male and 456 were female. Age was from 7 to 99 years (median, 62 years; mean, 60.96 ± 15.205 years). Most patients were married (611; 57.65%) and white (878; 82.83%). 55.66% of the patients had clear symptoms, of which the symptoms of A were 463 (43.68%) and B were 127 (11.98%). Out of 1060 PDL specimens, follicular lymphoma (FL) was observed in 436 (41.13%) of them, and diffuse large B-cell lymphoma (DLBCL) in 348 of the tumor specimens (32.83%) was observed. The majority of patients (949, 89.85%) had single tumor, and only 111 (10.47%) patients had multiple tumors. Among 1060 patients, 92 underwent surgery alone or associated with conservative treatment (chemotherapy alone, radiotherapy alone, chemotherapy + radiotherapy, or *Helicobacter pylori* eradication only), and the other 968 received conservative treatment.

Next, clinical and pathological features of 1060 PDLs were compared with those of 10573 PGLs and 3239 PSILs (Table 2). The results showed that there were no significant differences in age, gender, marital status, race, and other cancers between the surgery and conservative groups. However, primary site, Ann Arbor staging, symptoms, and histological type were significantly different between the two groups (all *P* < 0.05); that is, incidence of cancers with I stage or A symptoms was significantly higher in the conservative group compared to that in the surgery group.

The results showed that age, gender, symptom, and histological type were significantly different between PDLs and PGLs (all *P* < 0.05); that is, incidence of tumors with younger patients or more follicular lymphoma was significantly higher in the PDL group compared to that in the PGL group. The PDL group also showed younger patients, earlier Ann Arbor staging, more follicular lymphoma, and more surgery treatment in comparison with those of the PSIL group (all *P* < 0.05).

### 3.2. Survival and Prognostic Factors

**Figure 1:**
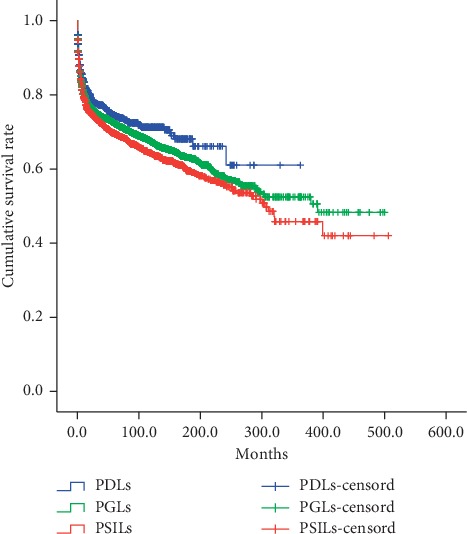
Comparison of DSS among PDLs, PGLs, and PSILs. The results showed that the DSS of PDLs were significantly better than those of PGLs and PSILs (10-year survival rate: 21.24% vs. 20.40%, *P*=0.027; 10-year survival rate: 21.24% vs. 16.79%, *P*=0.001). PDLs vs. PGLs: *P* < 0.05; PDLs vs. PSILs: *P* < 0.05. PGL, primary gastric lymphoma; PSIL, primary small intestinal lymphoma; PDL, primary duodenum lymphoma.

In order to analyze the prognosis among duodenum, gastric, and small intestinal lymphomas, survivals of 1060 PDLs were compared to those of 10573 PGLs and 3239 PSILs (Figure 1). The results showed that the DSS of PDLs were significantly better than those of PGLs and PSILs (10-year survival rate: 21.24% vs. 20.40%, *P*=0.027; 10-year survival rate: 21.24% vs. 16.79%, *P*=0.001).

Furthermore, univariate and multivariate analyses were performed to evaluate the prognosis of PDLs (Table 3). Age, gender, Ann Arbor staging, and histological type were regarded as independent prognostic factors for the DSS (all *P* < 0.05). Symptom was regarded as a significant risk factor for the DSS by univariate analysis (*P*=0.002), while it is not an independent prognostic factor for DSS by multivariate analysis.

### 3.3. Stratified Analysis

**Figure 2:**
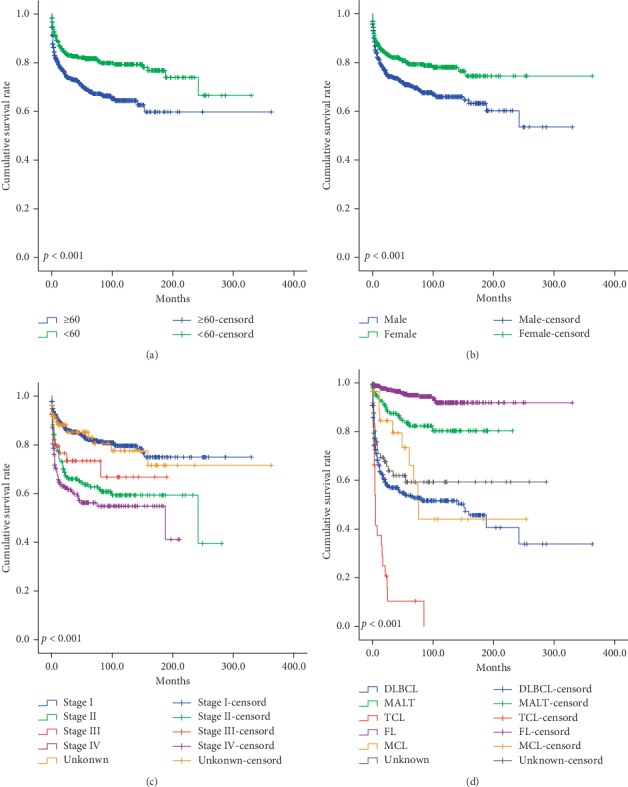
The stratified analysis according to (a) age, (b) gender, (c) Ann Arbor staging, and (d) histological type in the PDLs. Patients with <65 years, female, stage I, and FL were found to be significantly associated with good DSS. However, patients with ≥60 years, male, stage IV, and TCL were found to be significantly associated with poor DSS (all *P* < 0.05).

We showed stratified analysis according to several prognostic variables based on multivariate analyses (Figure 2). Patients with <65 years, female, stage I, and FL were found to be significantly associated with good DSS. However, patients with ≥60 years, male, stage IV, and TCL were found to be significantly associated with poor DSS (all *P* < 0.05).

**Figure 3:**
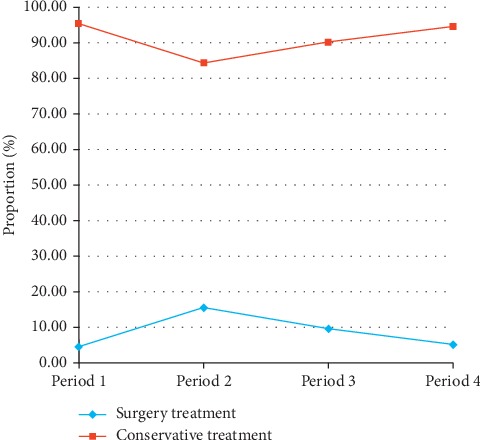
Trend of treatment modality (surgery vs. conservative treatment) to PGLs over the 18-year period from 1998 to 2015. The proportion of patients who received conservative treatment increased from 84.40% in period 2 to 94.74% in period 4, whereas patients who received surgical treatment gradually decreased from 15.60% in period 2 to 5.26% in period 4.

Figure 3 shows the changing trend of treatment modalities to PGL. The changing trends of treatment modalities to PDL were analyzed in 4 consecutive time periods: from 1998 to 2000 (period 1), from 2001 to 2005 (period 2), from 2006 to 2010 (period 3), and from 2011 to 2015 (period 4). The proportion of patients who received conservative treatment increased from 84.40% in period 2 to 94.74% in period 4, whereas patients who received surgical treatment gradually decreased from 15.60% in period 2 to 5.26% in period 4.

## 4. Discussion

To the best of our knowledge, the current study represented the largest number of PDLs. In this study, we summarized clinical and pathological features of 1060 cases of PDLs. We further analyzed prognosis of PDLs in comparison with that of PDLs and PSILs. It was found that tumors with younger patients or more follicular lymphoma was significantly higher in PDLs. In addition, PDLs had poorer prognosis compared to PGLs and PSILs. These observations indicate that surgery treatment may not play a role in improving survival in patients as compared to conservative treatment. Since 2000, the proportion of PDL patients undergoing surgery has declined.

We know that follicular lymphoma (FL) is primarily a nodal disease and primary FL of the gastrointestinal (GI) tract is rare [27]. However, the most common histological subtype is FL, followed by DLBCL among of PDLs [22]. Our study showed that the proportion of FL was the highest (44.13%) and significantly higher than that of stomach (2.23%) and small intestine (22.97%). Therefore, the predominance of the follicular histology in PDL was interesting. The high proportion of follicular lymphoma in duodenal lymphoma might be an important reason why the prognosis was better than that of the stomach and small intestine.

Our study showed that the mean age (60.96 ± 15.205) of patients with PDL was younger than that of the stomach and small intestine, and that the proportion of stage I was also higher than that of the stomach and small intestine. The duodenal anatomy site is special, the tumor growth space is small, and the patient presents the discomfort symptom earlier than the stomach and small intestinal. At the same time, EUS can not only clarify the lesions on the mucosal surface of the gastrointestinal tract but also understand the changes in the hierarchical structure of the gastrointestinal wall and its relationship with adjacent tissues and organs. It might relate to lower age and tumor staging.

Based on the assumption that gastrointestinal tract lymphoma is a localised disease, the surgical treatment was traditionally considered the cornerstone of the therapeutical strategy showing impressive results in terms of long survival. Nowadays, this approach has been extensively revised, and the management of gastrointestinal tract lymphoma is centred on systemic treatments such as chemo- and radiotherapy. From the EER data, the proportion of patients undergoing surgery gradually decreased from 2000 to 2015. From our study, the treatment of PDLs was also in line with the current treatment trend, but interestingly, the proportion of PDLs treated by surgery was lower than that of the stomach (9.64%) and the small intestine (35.13%), among which it was significantly lower than that of the small intestine. The reason may be that the duodenal lesion is mostly found in the descending segment [4–23], which has a complex anatomical structure and a small possibility of local resection, unlike the small intestine which can be directly resected, so conservative treatment is more preferred. Once a larger operation is performed, it is bound to cause complications and affect the quality of life. Meanwhile, multivariate analysis confirmed that the treatment modality was unrelated to DSS; that is, surgical treatment did not bring a survival advantage. The results were similar to previous reports (Table 4) [28–31] that the survival results of nonsurgical treatment were similar or even better than those of surgical treatment. Surgery, thus, is restricted to the treatment of complications such as occlusion, bleeding or perforation. Preventive surgery is sometimes advocated in bulky tumors, when rapid tumor necrosis secondary to chemo/radiotherapy may be associated with a high risk of life-threatening complications [28]. Surgery is also required for removal of residual disease after medical debulking [32]. Since the SEER database does not list the complications, this paper cannot discuss the complications.

Although there was no statistically significant difference in survival by treatment modalities in the multivariate analysis, there are other multiple factors that contribute to survival. In previous studies, female, low-grade histology and good PS have been reported to be associated with high OS. However, age >60 years, advanced stage, poor performance status (PS), and elevated lactic dehydrogenase (LDH) were associated with poor outcome [3, 32–34]. In our study, age, gender, Ann Arbor staging, and histological type retained independent prognostic factors in the multivariate analysis. Patients with <65 years, female, stage I , and FL were found to be significantly associated with good DSS. LDH and PS are not mentioned in the SEER database, so statistical analysis cannot be made in this paper.

Although it is an excellent resource for comparative outcome analysis for all malignancies involving the gastrointestinal tract, SEER has its limitations. Since the database provides passive follow-up for its registered cases, incomplete data reporting remains a problem. First, much information could not be obtained from the SEER database, such as PS and LDH. Second, the SEER database also did not describe postoperative complications and quality of life score, so we were unable to assess the complications and quality of life associated with surgery.

## Figures and Tables

**Table 1:** Demographics and characteristics of PDLs.

Clinicopathologic features	Number of assessable patients (%)
Age (years)	
Mean ± SD	60.96 ± 15.205
≥60	578 (54.53)
<60	482 (45.47)
Gender	
Male	604 (56.98)
Female	456 (43.02)
Marital status	
Married	611 (57.65)
Unmarried	349 (32.92)
Unknown	100 (9.43)
Race	
White	878 (82.83)
Black	69 (6.51)
Others	93 (8.77)
Unknown	20 (1.89)
Ann Arbor staging	
I	564 (53.21)
II	182 (17.17)
III	40 (3.77)
IV	173 (16.32)
Unknown	101 (9.53)
Symptoms	
A	463 (43.68)
B	127 (11.98)
Unknown	470 (44.34)
Histological type	
DBCLC	348 (32.83)
MALT	146 (13.77)
T-cell	28 (2.64)
FL	436 (41.13)
MCL	29 (2.74)
Unknown	73 (6.89)
Combined with other cancers	
Yes	111 (10.47)
No	949 (89.53)
Treatment modality	
Only surgery	41 (3.87)
Surgery + conservative	51 (4.81)
Conservative	968 (91.32)

DLBCL = diffuse large B-cell lymphoma; ETCL = T-cell lymphoma; FL = follicular lymphoma; MALT = mucosa-associated lymphoid tissue; MCL = mantle cell lymphoma.

**Table 2:** Comparison of clinicopathological parameters among PSLs, PGLs, and PSILs.

Clinicopathologic features	PDLs	PGLs		PSILs	
	*n* = 1060	*n* = 10573	*P* value	*n* = 3239	*P* value
Age (years)					
Mean ± SD	60.96 ± 15.205	66.08 ± 14.957	<0.001	62.41 ± 16.779	0.013
≥60	578 (54.53)	7205 (68.15)	<0.001	1927 (59.49)	0.004
<60	482 (45.47)	3368 (31.85)		1312 (40.51)	
Gender			0.016		0.065
Male	604 (56.98)	5617 (53.13)		1931 (59.62)	
Female	456 (43.02)	4956 (46.87)		1308 (40.38)	
Ann Arbor staging			0.071		<0.001
I	564 (53.21)	5616 (53.12)		1239 (38.25)	
II	182 (17.17)	1541 (14.58)		1047 (32.32)	
III	40 (3.77)	482 (4.56)		143 (4.41)	
IV	173 (16.32)	1731 (16.37)		567 (17.52)	
Unknown	101 (9.53)	1203 (11.37)		243 (7.50)	
Symptoms			<0.001		<0.001
A	463 (43.68)	3260 (30.83)		1174 (36.25)	
B	127 (11.98)	1313 (12.42)		432 (13.33)	
Unknown	470 (44.34)	6000 (56.75)		1633 (50.42)	
Histological type			<0.001		<0.001
DLBCL	348 (32.83)	5168 (48.88)		1778 (54.89)	
MALT	146 (13.77)	4323 (40.89)		265 (8.18)	
T-cell	28 (2.64)	78 (0.74)		185 (5.71)	
FL	436 (41.13)	236 (2.23)		744 (22.97)	
MCL	29 (2.74)	131 (1.24)		51 (1.57)	
Unknown	73 (6.89)	637 (6.02)		216 (6.68)	
Treatment modality			0.38		<0.001
Only surgery	41 (3.87)	401 (3.79)		928 (28.65)	
Surgery + conservative	51 (4.81)	619 (5.85)		1173 (36.22)	
Conservative	968 (91.32)	9553 (90.36)		1138 (35.13)	

DLBCL = diffuse large B-cell lymphoma; ETCL = T-cell lymphoma; FL = follicular lymphoma; MALT = mucosa-associated lymphoid tissue; MCL = mantle cell lymphoma.

**Table 3:** Univariate and multivariate analysis for DSS in the PGLs.

Characteristics	Univariate analysis			Multivariate analysis		
	HR	95% CI	*P* value	HR	95% CI	*P* value
Age (years)	1.026	1.017–1.035	<0.001	1.027	1.018–1.036	<0.001
Gender	0.636	0.491–0.824	0.001	0.633	0.487–0.823	0.001
Marital status	1.136	0.950–1.358	0.162			
Symptom	1.239	1.083–1.417	0.002			
Ann Arbor staging	1.189	1.099–1.286	<0.001	1.202	1.107–1.306	<0.001
Histological type	0.696	0.638–0.759	<0.001	0.718	0.656–0.786	<0.001
Combined with other cancers	0.724	0.483–1.087	0.119			
Treatment modality	1.158	0.993–1.349	0.061	1.034	0.906–1.182	0.618

**Table 4:** Previously reported cases of PDLs.

Reference	Num	Age	Sex	Location	Type	Stage	CD markers	Surgery	Conservation	Follow-up
Zheng et al. [4]	1	58	M	——	MCL	——	CD20, CD21, CD5, BCL-2	None	None	——
Linnik et al. [5]	1	51	M	——	DLBCL	——	CD20, CD45, BCL2, BCL6	None	Chemotherapy	60 mo/L
Iwamuro et al. [6]	2	52	M	Descendant duodenum	FL	IV	CD20, CD10, BCL2	——	——	——
		96	F	Descendant duodenum	FL	IV	CD20, CD10, BCL2	——	——	——
Mejia et al. [7]	1	56	M	Papilla	FL	——	CD-20, CD10, BCL-2, BCL-6	None	R	——
Iwamuro et al. [8]	1	60	M	Descendant duodenum	FL	IV	CD20, CD10, BCL2	None	Bendamustine and R	——
Trivedi et al. [9]	1	36	F	Ampulla of Vater	DLBCL	——	CD20	YES	Chemotherapy	2 y/L
Tari et al. [10]	1	66	F	Ampulla of Vater	FL	II2	CD20, CD10, BCL-2	None	None	——
Du et al. [11]	1	65	M	Descendant duodenum	DLBCL and TCL	——	CD20, CD3, CD45	YES	R-CHOP	30 mo/R
Cho et al. [12]	1	68	M	Duodenal bulb	MALT	——	——	YES	——	——
Kondo et al. [13]	1	78	F	Ampulla of Vater	DLBCL	——	CD20, CD10, CD79a, BCL-2	YES	R-CHOP	19 mo/NR
Nakase et al. [14]	1	57	F	Papilla	FL	I	CD10, Bcl-2	None	None	1 mo/NR
Born et al. [15]	1	75	F	——	FL	——	——	None	None	——
Woo et al. [16]	1	71	F	Descendant duodenum	MALT	EII2	CD20	None	CVP	1 y/NR
Chim et al. [17]	1	73	M	Ampulla of Vater	DLBCL	II1	CD20, BCL-6	None	CHOP	Died due to COPD
Jabr [18]	1	71	F	Ampulla of Vater	DLBCL	——	CD20, CD10, CD45, BCL-2	None	Chemotherapy	——
Zenda et al. [19]	1	49	F	Papilla	FL	I	CD20, CD10, CD79a, BCL-2	None	R-CHOP	——
Yildirim et al. [20]	3	33	M	Ampulla of Vater	DLBCL	——	CD20	None	CHOP	1 y/NR
		24	M	Ampulla of Vater	DLBCL	——	CD20, CD45	None	CHOEP	Died due to sepsis and multiorgan failure
		38	M	Ampulla of Vater	DLBCL	——	CD20, LCA	None	CHOP	Died due to suspected perforation
Isomoto et al. [21]	1	46	M	Ampulla of Vater	MALT	IE	BCL-2	None	Radiation	4 y/NR
Nadal et al. [22]	1	55	M	Ampulla of Vater	FL	——	CD10, Bcl-2	None	CHOP	2 y/NR
Ventrucci et al. [23]	1	65	F	Ampulla of Vater	MALT	——	CD20, CD79a, BCL-2	None	CVP	15 mo/NR

## Data Availability

No additional data are available.

## Abbreviations

CHOP: cyclophosphamide, doxorubicin, vincristine, and prednisolone
COPD: chronic obstructive pulmonary disease
CVP: cyclophosphamide, vincristine, and prednisone
D: died
DLBCL: diffuse large B-cell lymphoma
DSS: disease-specific survival
ETCL: enteropathy-type T-cell lymphoma
F: female
FL: follicular lymphoma
JGCA: Japanese Gastric Cancer Association
L: live
LDH: lactic dehydrogenase
M: male
MALT: mucosa-associated lymphoid tissue
MCL: mantle cell lymphoma
NHL: non-Hodgkin's lymphoma
NR: no recurrence
PDL: primary duodenum lymphoma
PGIL: primary gastrointestinal
PGLs: primary gastric lymphoma
PS: performance status
PSILs: primary small intestinal lymphoma
R: recurrence
R-CHOP: rituximab with CHOP
SEER: Surveillance, Epidemiology, and End Results
